# Supplementary material for: Shared proteomic landscape between arteriosclerosis and cardiovascular endpoints: a Mendelian randomization and observational study integrating AlphaFold3 for structural prediction
Source: Cardiovasc Res. 2026 May 14;122(9):1159–75. doi: 10.1093/cvr/cvag095 (PMC13307572; doi:10.1093/cvr/cvag095)
Supplement: cvag095_Supplementary_Data [file cvag095_supplementary_data.zip › Supplemental Figures.pdf]

Supplemental Figures

Shared proteomic landscape between arteriosclerosis and cardiovascular endpoints: a Mendelian randomization and observational study integrating AlphaFold3 for structural prediction

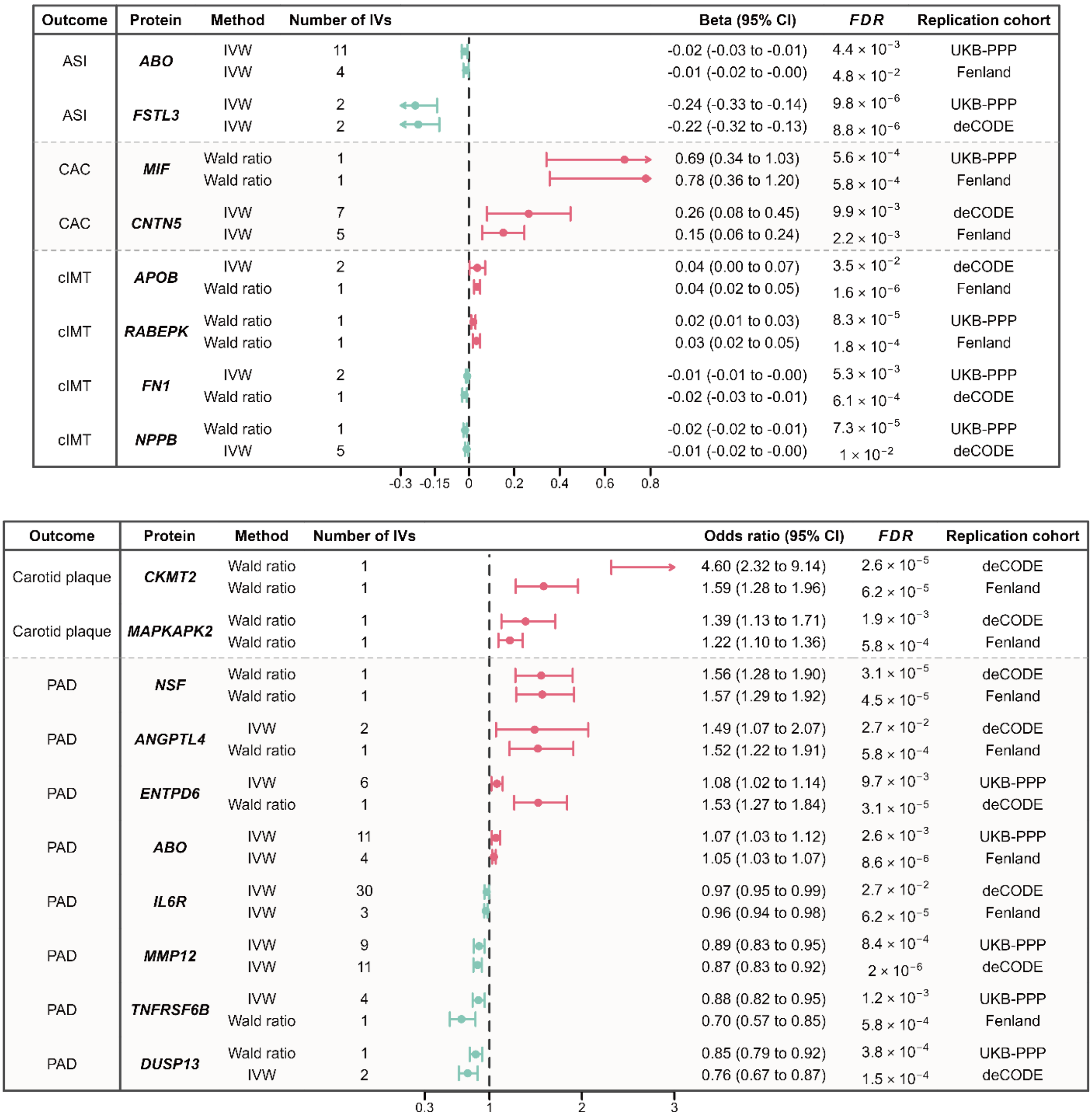

Supplemental Figure 1. Candidates for arteriosclerotic/atherosclerotic markers that replicated in an independent cohort in Mendelian randomization analysis. The upper panel shows continuous outcomes while the lower panel shows dichotomous outcomes; All estimates correspond to a one-standard-deviation increase in protein levels; ASI, arterial stiffness index; CAC, coronary artery calcification; cIMT, carotid artery intima-media thickness; PAD, peripheral artery disease; IVs, instrumental variables.

Outcome	Protein	Method	Number of IVs	Odds ratio (95% CI)	*FDR*	Replication cohort
Any stroke	***MMP12***	IVW	8	0.94 (0.90 to 0.98)	2.5×10^{-3}	UKB-PPP
		IVW	2	0.92 (0.90 to 0.94)	1.9×10^{-9}	Fenland
Any ischaemic stroke	***MMP12***	IVW	8	0.94 (0.89 to 0.98)	1.1×10^{-2}	UKB-PPP
		IVW	2	0.92 (0.89 to 0.94)	2.6×10^{-9}	Fenland
Cardioembolic stroke	***ABO***	IVW	9	1.10 (1.03 to 1.17)	3.5×10^{-3}	UKB-PPP
		IVW	4	1.06 (1.03 to 1.10)	4.2×10^{-4}	Fenland
Large-artery stroke	***ABO***	IVW	8	1.18 (1.08 to 1.28)	6.7×10^{-4}	UKB-PPP
		IVW	4	1.09 (1.04 to 1.14)	4.3×10^{-4}	Fenland
Large-artery stroke	***MMP12***	IVW	7	0.81 (0.68 to 0.98)	3×10^{-2}	UKB-PPP
		IVW	5	0.79 (0.70 to 0.90)	2.1×10^{-4}	deCODE
CAD	***APOB***	IVW	2	1.60 (1.04 to 2.47)	3.3×10^{-2}	deCODE
		Wald ratio	1	1.53 (1.38 to 1.69)	1.5×10^{-15}	Fenland
CAD	***ANGPTL4***	Wald ratio	1	1.55 (1.35 to 1.78)	9.7×10^{-10}	deCODE
		Wald ratio	1	1.54 (1.34 to 1.76)	1.2×10^{-9}	Fenland
CAD	***ABO***	IVW	10	1.05 (1.02 to 1.08)	3.2×10^{-3}	UKB-PPP
		IVW	4	1.02 (1.01 to 1.03)	2.1×10^{-3}	Fenland
CAD	***IL6R***	IVW	16	0.96 (0.94 to 0.98)	8.5×10^{-7}	deCODE
		IVW	3	0.97 (0.96 to 0.98)	1.1×10^{-9}	Fenland
CAD	***DUSP13***	Wald ratio	1	0.91 (0.87 to 0.95)	8.5×10^{-5}	UKB-PPP
		Wald ratio	1	0.85 (0.79 to 0.92)	5.1×10^{-5}	deCODE
CAD	***FN1***	IVW	2	0.90 (0.83 to 0.98)	1.1×10^{-2}	UKB-PPP
		Wald ratio	1	0.77 (0.71 to 0.83)	5.7×10^{-10}	deCODE
Myocardial infarction	***APOB***	IVW	2	1.60 (1.27 to 2.01)	7.8×10^{-5}	deCODE
		Wald ratio	1	1.44 (1.25 to 1.66)	1.2×10^{-6}	Fenland
Myocardial infarction	***ANGPTL4***	Wald ratio	1	1.57 (1.30 to 1.89)	3.7×10^{-6}	deCODE
		Wald ratio	1	1.55 (1.29 to 1.86)	3.3×10^{-6}	Fenland
Myocardial infarction	***ABO***	IVW	8	1.07 (1.04 to 1.10)	1×10^{-6}	UKB-PPP
		IVW	4	1.05 (1.03 to 1.06)	1.2×10^{-10}	Fenland
Myocardial infarction	***FN1***	Wald ratio	1	0.77 (0.69 to 0.85)	1.3×10^{-6}	deCODE
		Wald ratio	1	0.64 (0.54 to 0.76)	2.8×10^{-7}	Fenland

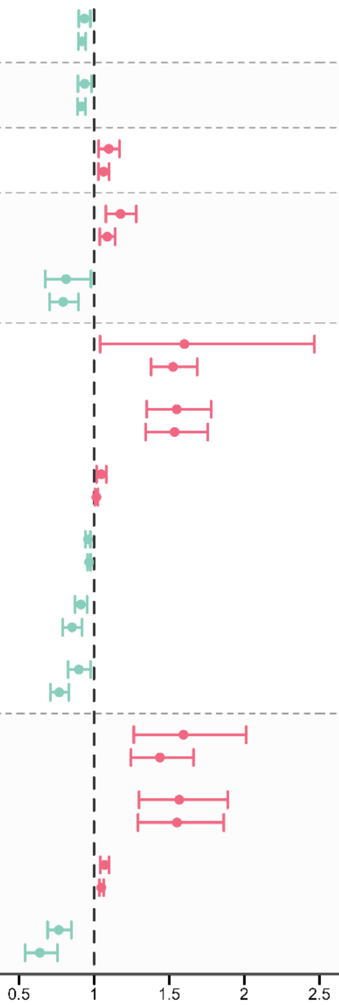

Supplemental Figure 2. Candidates for cardiovascular events that replicated in an independent cohort in Mendelian randomization analysis. All estimates correspond to a one-standard-deviation increase in protein levels; CAD, coronary artery disease; IVs, instrumental variables.

Bulk tissue gene expression for APOB (ENSG00000084674.15)

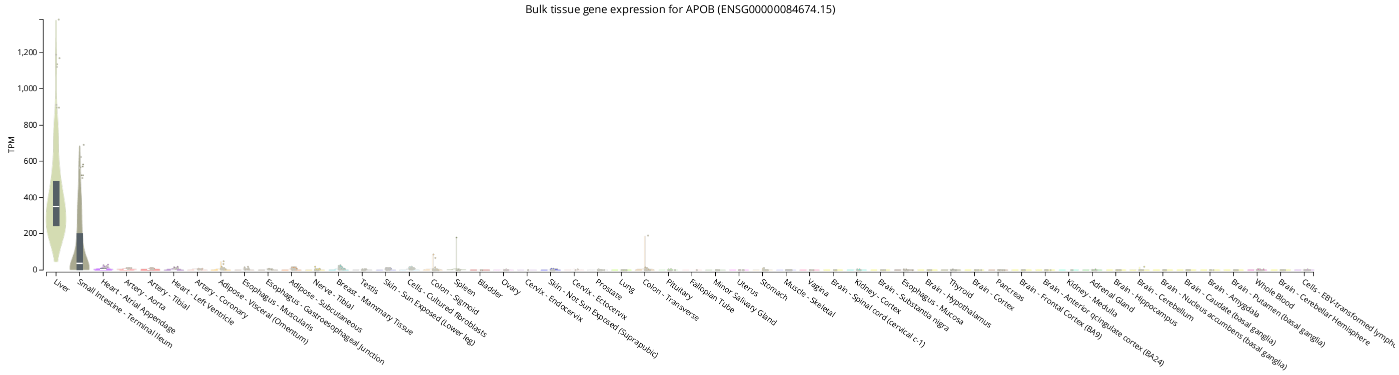

Bulk tissue gene expression for DUSP13 (ENSG00000079393.21)

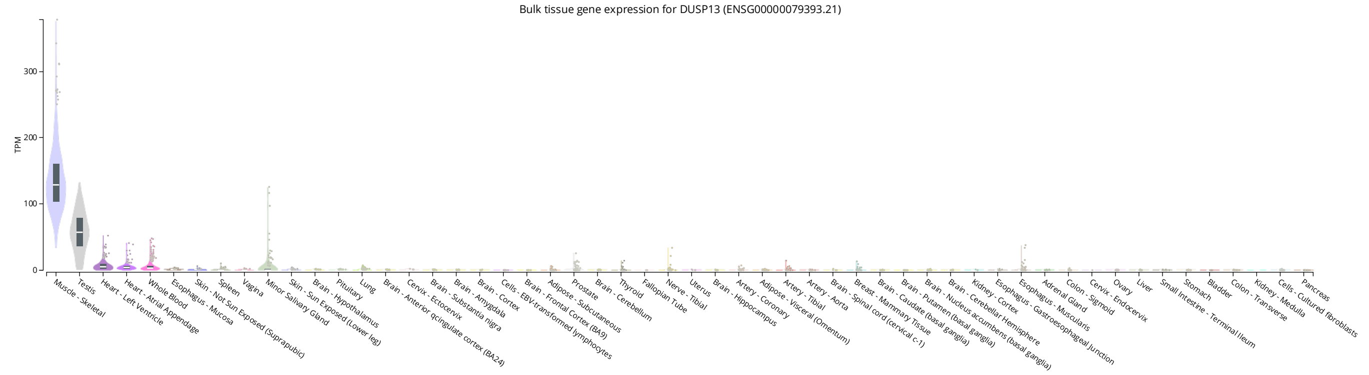

Bulk tissue gene expression for FN1 (ENSG00000115414.21)

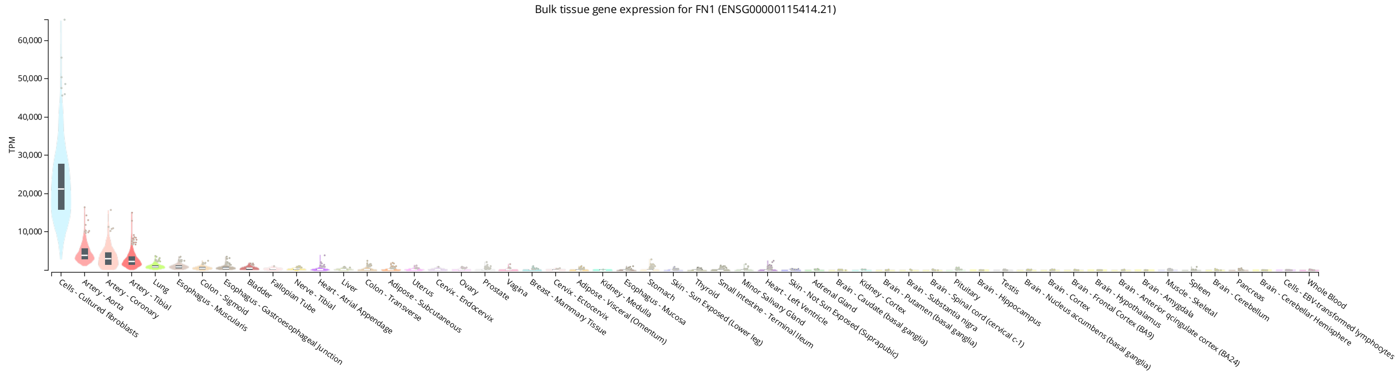

Bulk tissue gene expression for ZPR1 (ENSG00000109917.11)

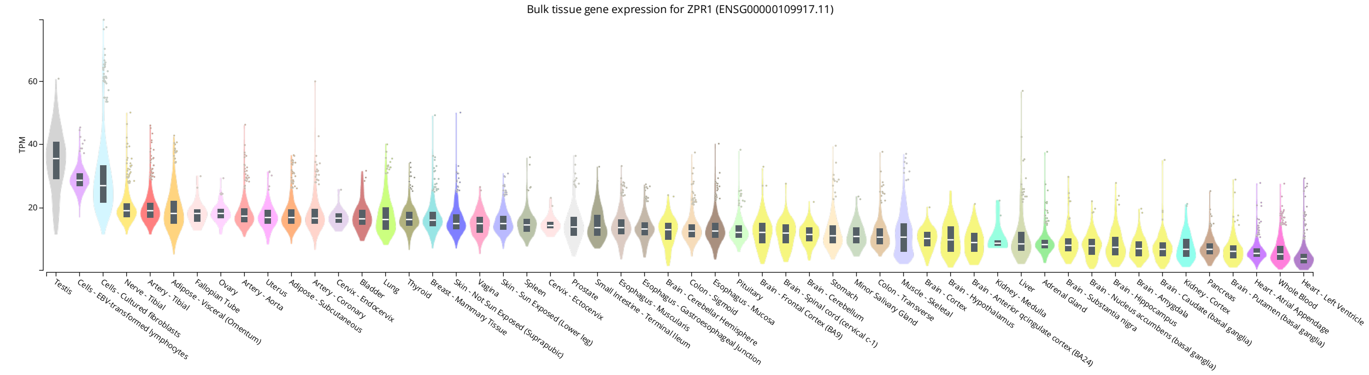

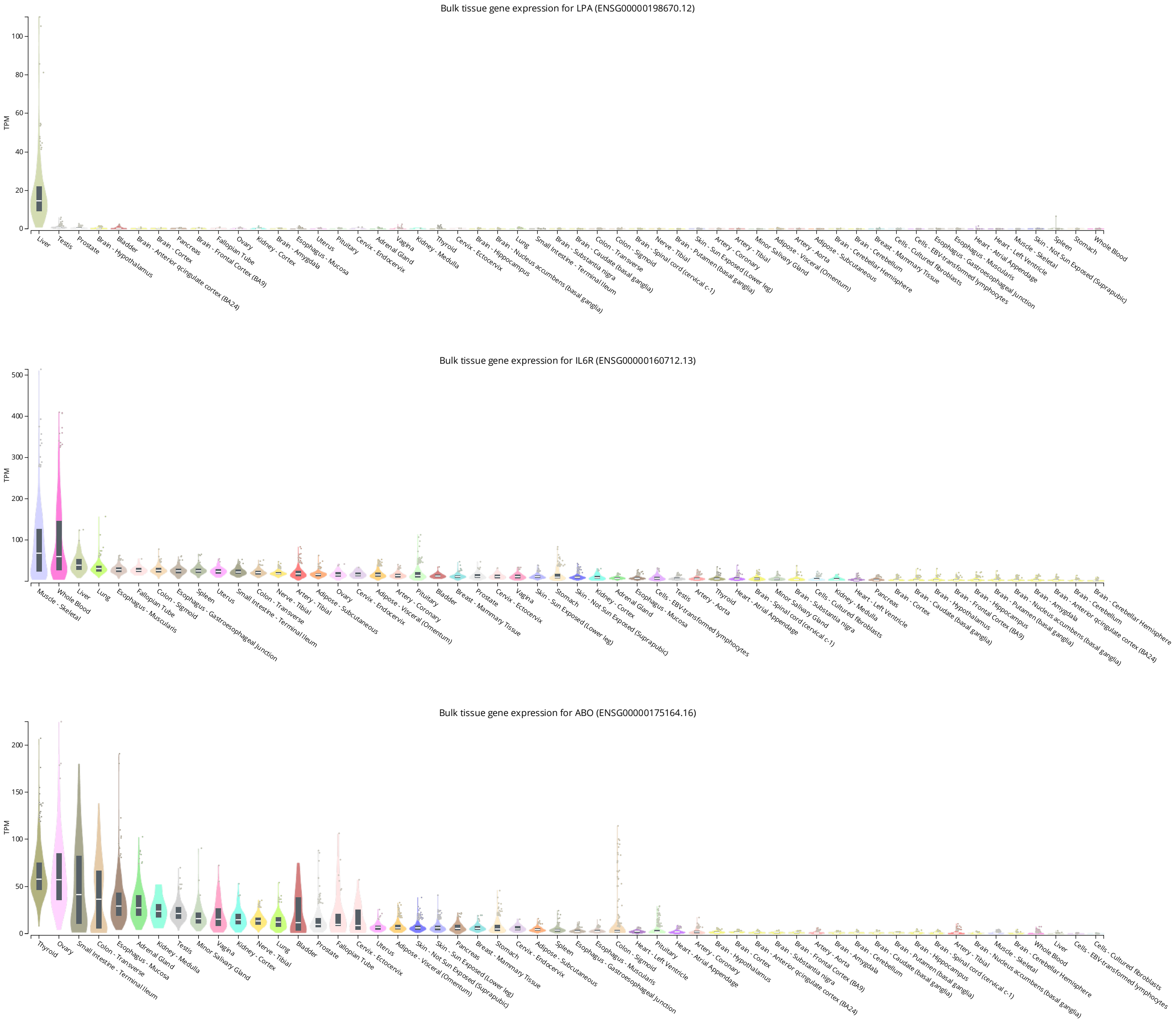

Supplemental Figure 3. Look up of gene expression level from GTEx V10 for the prioritized candidates.

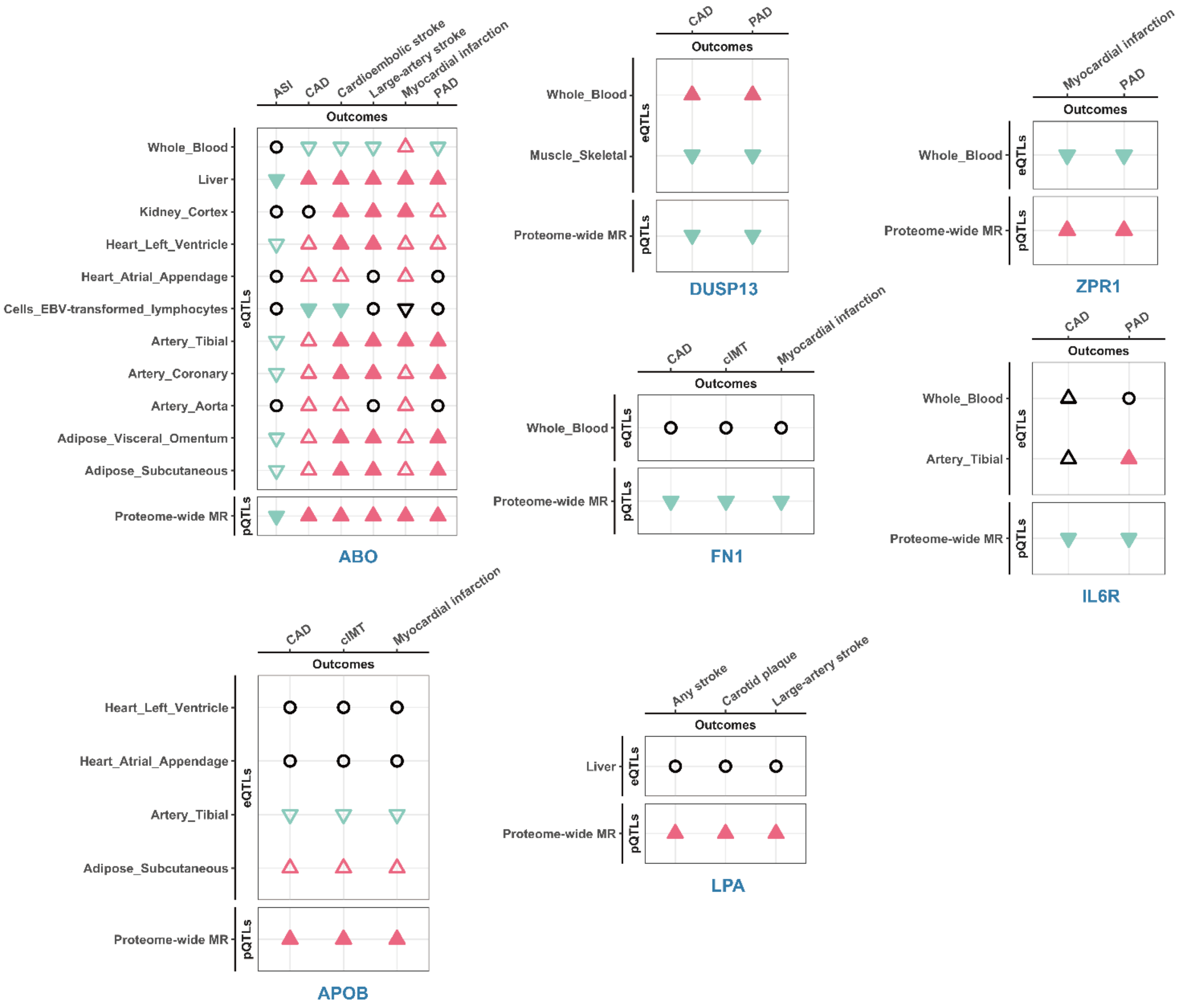

Supplemental Figure 4. Results from transcriptome-wide analysis applying SMR and HEIDI. The Y-axis displays the names of tissues in which gene expression levels were proxied by eQTLs as the exposure; the lower panels labels as pQTLs show the results from the previous proteome-wide MR analysis. The X-axis displays the names of outcomes (arteriosclerotic/atherosclerotic markers and cardiovascular events). Triangle: nominal significant; Coloured triangle: FDR significant; Filled triangle: pass HEIDI test; Hollow triangle: does not pass HEIDI test; Circle: not significant; The direction of the triangle represents the direction of the association. ASI, arterial stiffness index; cIMT, carotid artery intima-media thickness; PAD, peripheral artery disease; CAD, coronary artery disease.

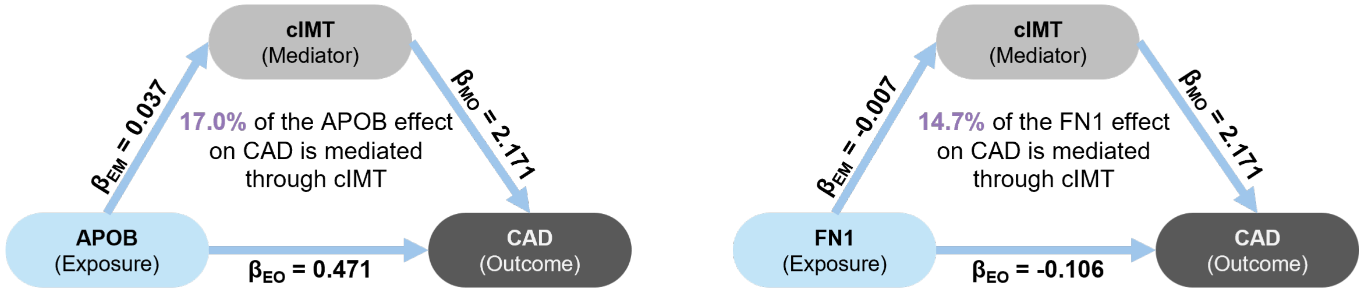

Supplemental Figure 5. Diagrams showing proportion of protein effect on CAD and stroke outcomes through cIMT. β_{EM}, effects of exposure on mediator; β_{EO}, effects of exposure on the outcome; β_{MO}, effects of mediator on the outcome; cIMT, carotid artery intima-media thickness; CAD, coronary artery disease.

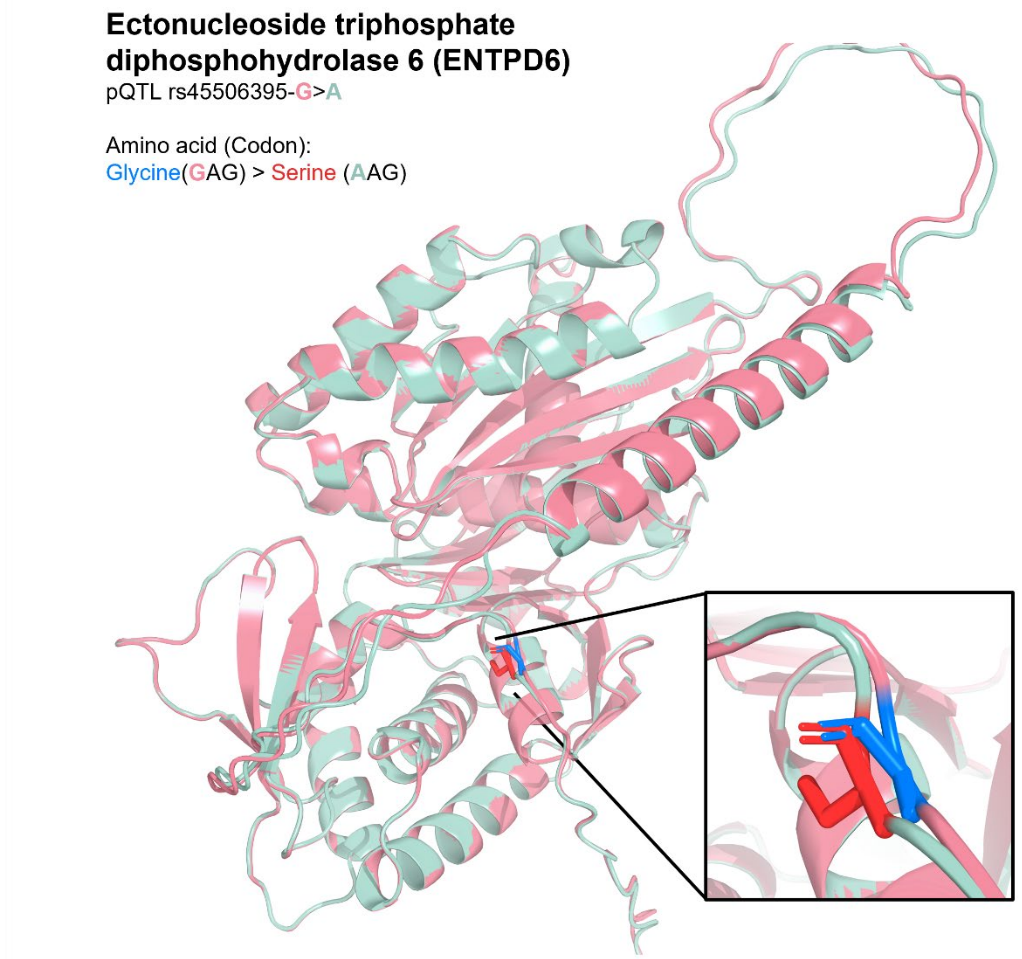

Supplemental Figure 6. Predicted structural alterations of ENTPD6 resulted from the missense variant rs45506395. The 3D structure of wild-type protein (reference allele) and mutated protein (alternative allele) were coloured in pink and cyan, respectively. The amino acid encoded by reference codon and alternative codon were highlighted in stick representation and coloured in blue and red, respectively.

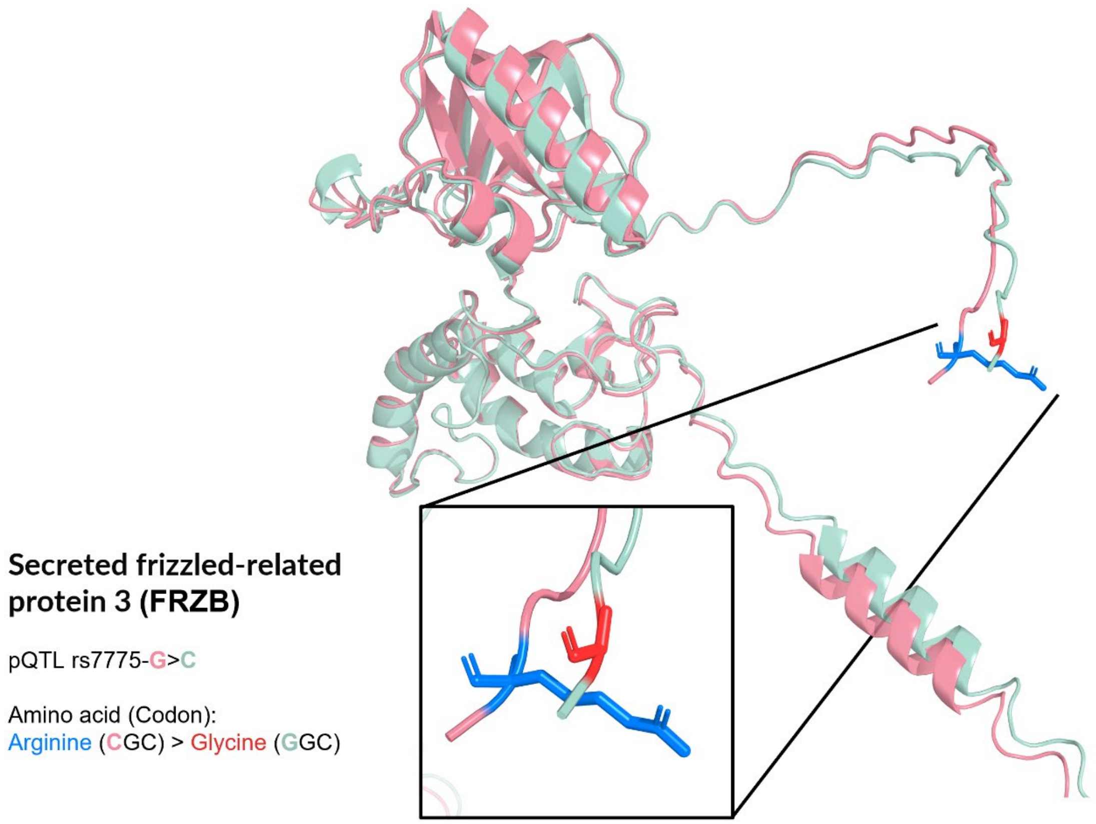

Supplemental Figure 7. Predicted structural alterations of FRZB resulted from the missense variant rs7775. The 3D structure of wild-type protein (reference allele) and mutated protein (alternative allele) were coloured in pink and cyan, respectively. The amino acid encoded by reference codon and alternative codon were highlighted in stick representation and coloured in blue and red, respectively.

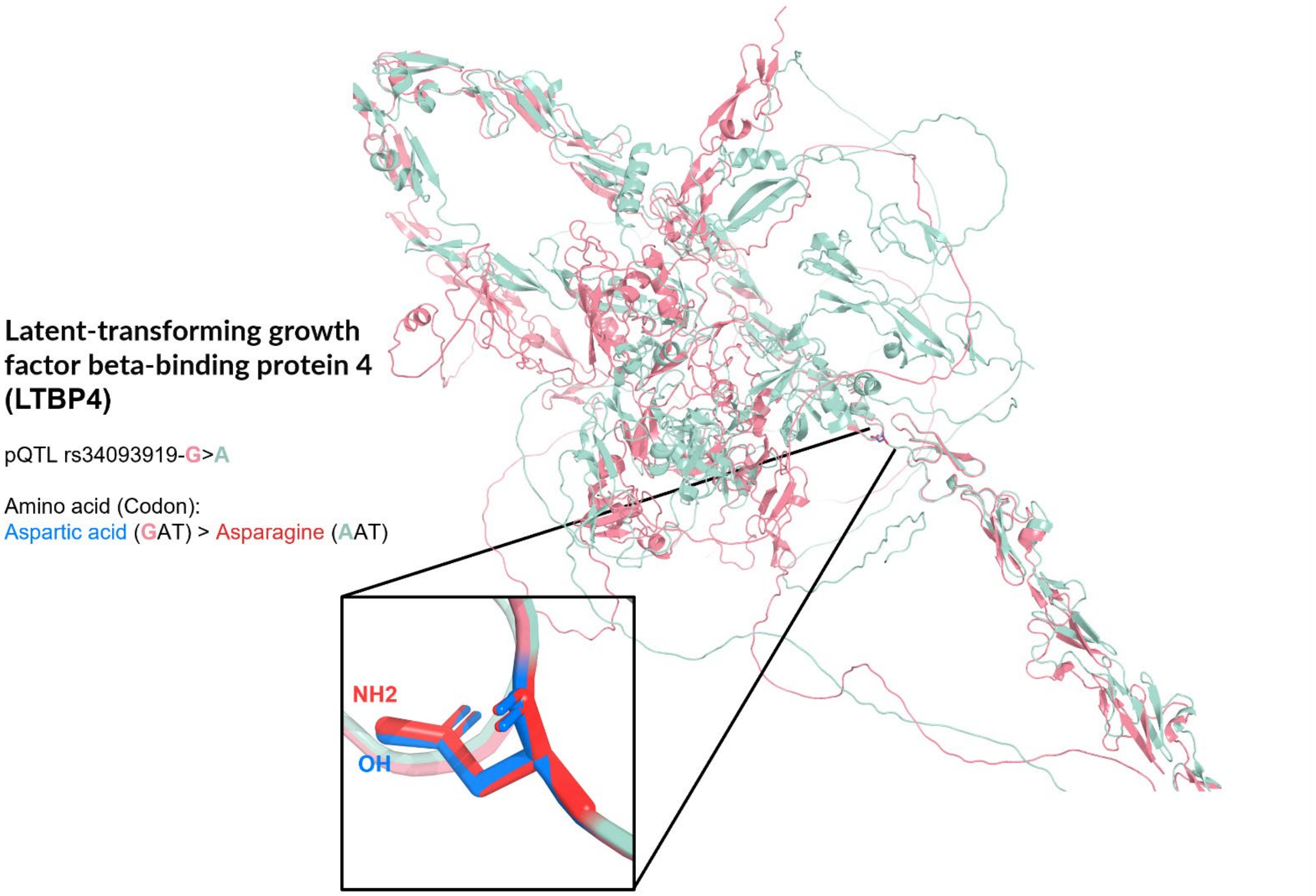

Supplemental Figure 8. Predicted structural alterations of LTBP4 resulted from the missense variant rs34093919. The 3D structure of wild-type protein (reference allele) and mutated protein (alternative allele) were coloured in pink and cyan, respectively. The amino acid encoded by reference codon and alternative codon were highlighted in stick representation and coloured in blue and red, respectively.

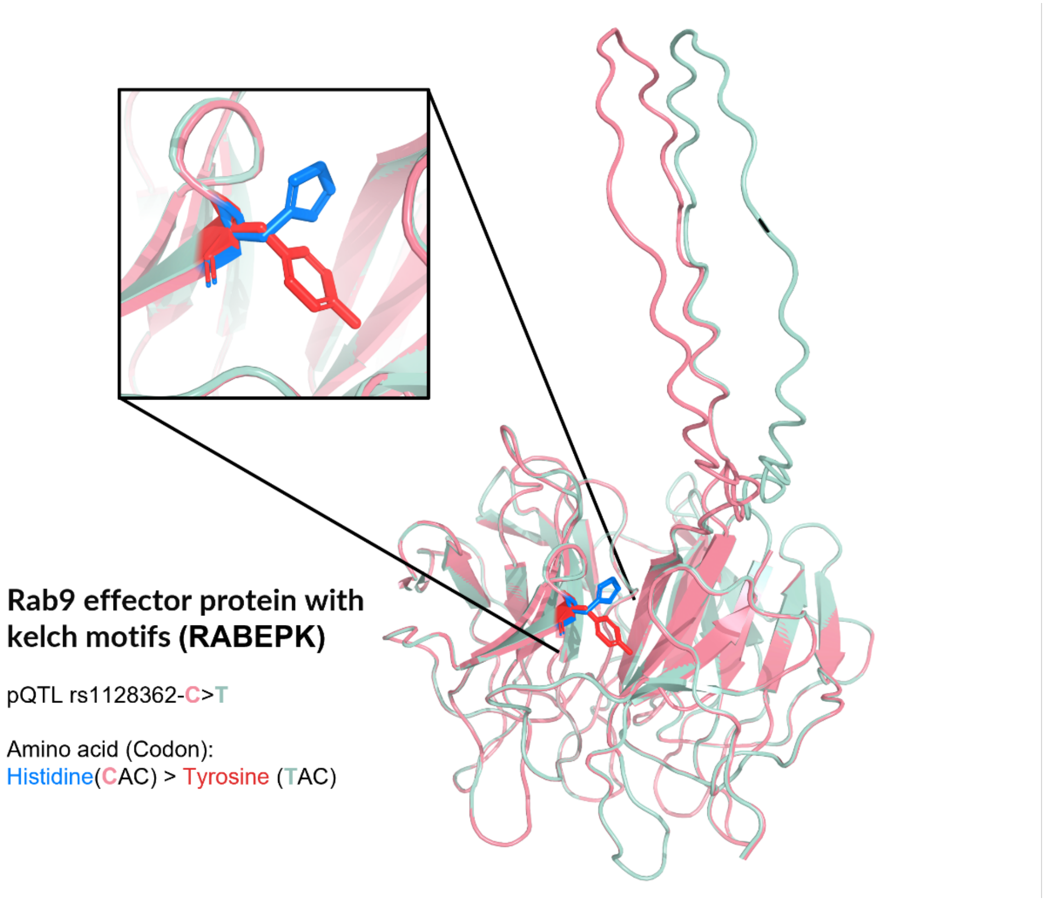

Supplemental Figure 9. Predicted structural alterations of RABEPK resulted from the missense variant rs1128362. The 3D structure of wild-type protein (reference allele) and mutated protein (alternative allele) were coloured in pink and cyan, respectively. The amino acid encoded by reference codon and alternative codon were highlighted in stick representation and coloured in blue and red, respectively.

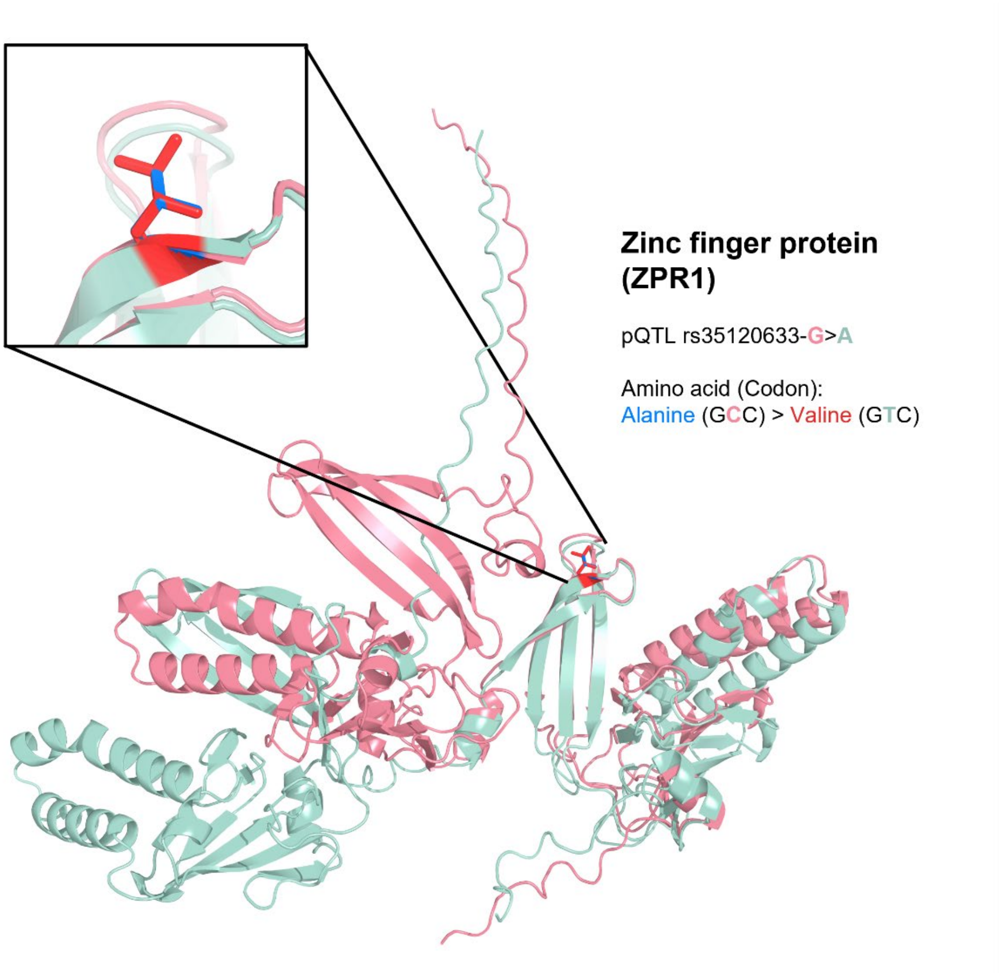

Supplemental Figure 10. Predicted structural alterations of ZPR1 resulted from the missense variant rs35120633. The 3D structure of wild-type protein (reference allele) and mutated protein (alternative allele) were coloured in pink and cyan, respectively. The amino acid encoded by reference codon and alternative codon were highlighted in stick representation and coloured in blue and red, respectively.
